# The pattern of Social Cognition impairment: a 1‐year follow‐up study in Brazilians with Alzheimer´s Disease

**DOI:** 10.1002/alz.095244

**Published:** 2025-01-09

**Authors:** Tatiana Belfort, Marcela Moreira Lima Nogueira, Isabel Lacerda, Michelle Brandt, Aline Lucena, Rogeria Rangel, Julia Gaigher, Felipe de Oliveira Silva, Natalie Souza, Marcia C. N. Dourado

**Affiliations:** ^1^ Federal University of Rio de Janeiro, Rio de Janeiro Brazil; ^2^ Federal University of Rio de Janeiro, Rio de Janeiro, Rio de Janeiro Brazil

## Abstract

**Background:**

Social Cognition (SC) is the ability to recognize and understand socially relevant stimuli, which contributes to an adequate social decision. Different neurological conditions may affect this cognitive domain, especially Alzheimer’s disease (AD). SC impairments are commonly associated with the progression of the disease and contribute to the dependence of people with AD, influencing their functional disability and the burden on family members and caregivers.The study aims to investigate longitudinally the relationship between SC and cognitive and clinical variables. We also examined the relation of the different SC predictors from three perspectives: people with AD, caregivers of people with AD, and discrepancy analysis.

**Method:**

In baseline (M1), 137 dyads (people with AD and their caregivers) were assessed, and a new assessment was realized after one year (M2). Were excluded 58 dyads during the follow‐up. The study was concluded with 79.

**Result:**

SC impairments showed a more stable pattern, while cognitive functions declined, which is consistent with disease progression and in line with the literature. Regarding SC predictors, SC was associated with cognition for people with AD at both time points. For caregivers, besides cognition, other predictors included reduced functional abilities and quality of life in people with AD.

**Conclusion:**

Our findings suggest that Social Cognition in AD may be influenced more by emotional processing than cognitive impairment, presenting a more stable pattern longitudinally. A more comprehensive approach, investigating different points of view in SC, enables a more global understanding, significantly contributing to better and more targeted treatment for the patient. This is particularly relevant when considering a domain that includes subjective factors such as values and beliefs, which evoke social, cultural, and contextual factors.

